# Preoperative information for ICU patients to reduce anxiety during and after the ICU-stay: protocol of a randomized controlled trial [NCT00151554]

**DOI:** 10.1186/1472-6955-5-4

**Published:** 2006-03-08

**Authors:** Almuth Berg, Steffen Fleischer, Michael Koller, Thomas R Neubert

**Affiliations:** 1 University Hospital Gieβen and Marburg, Location Marburg, Germany; 2 University Hospital Regensburg, Germany

## Abstract

**Background:**

According to current evidence and psychological theorizing proper information giving seems to be a promising way to reduce patient anxiety. In the case of surgical patients, admission to the intensive care unit (ICU) is strongly associated with uncertainty, unpredictability and anxiety for the patient. Thus, ICU specific information could have a high clinical impact. This study investigates the potential benefits of a specifically designed ICU-related information program for patients who undergo elective cardiac, abdominal or thoracic surgery and are scheduled for ICU stay.

**Methods/Design:**

The trial is designed as a prospective randomized controlled trial including an intervention and a control group. The control group receives the standard preparation currently conducted by surgeons and anesthetists. The intervention group additionally receives a standardized information program with specific procedural, sensory and coping information about the ICU.

A measurable clinical relevant difference regarding anxiety will be expected after discharge from ICU. Power calculation (α = 0.05; β = 0.20; Δ = 8.50 score points) resulted in a required sample size of N = 120 cardiac surgical patients (n = 60 vs. n = 60). Furthermore, N = 20 abdominal or thoracic surgical patients will be recruited (n = 10 vs. n = 10) to gain insight to a possible generalization to other patient groups.

Additionally the moderating effect of specific patient attributes (need for cognition, high trait anxiety) will be investigated to identify certain patient groups which benefit most.

**Discussion:**

The proposed study promises to strengthen evidence on effects of a specific, concise information program that addresses the information needs of patients scheduled for ICU stay.

## Background

Technological advances during the last decades in intensive care medicine made it possible to treat an increasing number of high risk surgical patients. Nevertheless, many patients awaiting elective surgery experience diffuse anxiety and a lack of subjective predictability regarding their stay on the intensive care unit. The psychological elements that become salient in the perioperative situation can be characterized as loss of control, irritation and uncertainty. These psychological elements are also known for contributing to postoperative complications such as delayed wound healing and an increased intake of sedatives and analgesics [1]. In addition, the strange and unfamiliar situation on the ICU in combination with sleep deprivation and ICU-noises is closely related with the incidence of a postoperative delirium also called ICU-Syndrome [2,3]. In a preliminary study it was shown that only 16% of the asked patients felt sufficiently informed and prepared for their ICU-stay [4].

According to the psychological theory of control proper preoperative information giving seems a promising way to reduce ICU-related anxiety [5-7].

A proper information program would inform the patient about the aims, prospects and the specific elements of the scheduled ICU stay. Thus, the patient learns that he/she is about to undergo a time-limited episode and that all is done in the best of his/her interest. This information together with learning about ways how to communicate in the case of specific needs gives the patient back a sense of control in this communicative difficult situation on the ICU.

A number of studies indicate that surgical patients profit from preoperative information with regard to various outcomes such as anxiety, pain and length of stay [8-17]. Nevertheless, the evidence that information regarding ICU-specific issues is beneficial is scarce [18-20] and a recent review concludes that randomised studies in this area are called for [18].

Therefore the aim of our study is to investigate the benefits of a standardized information program that prepared the patient for a scheduled ICU stay following an operation. This information program is tested in a randomized controlled trial, in which the control group receives the standard preoperative information and the intervention group the standard preoperative information plus the ICU-specific information.

The hypothesis of our study is straightforward: The information program reduces the experience of anxiety and other unpleasant emotions and cognitions that are related to the ICU stay. Furthermore we expect that the information program has a positive influence on quality of life and satisfaction with the hospital 3 months postoperatively.

## Methods/Design

This trial is based on a non-blinded, randomized controlled study design. The control group receives the routine treatment and information by the surgeon and the anesthetist, the experimental group additionally receives an ICU-specific information program on the day before surgery.

### Inclusion and exclusion criteria

The study is conducted in cooperation with the Department of Heart Surgery and the Department of Visceral, Thoracic and Vascular Surgery at the University Hospital Gieβen and Marburg, Location Marburg, Germany. In principle, all patients of these surgical departments that await elective surgery with a planned subsequent admission to the intensive care unit are eligible for this study. Mainly patients that undergo coronary artery bypass surgery and valve replacement or high risk patients undergoing thoracotomy, gastrectomy, pancreatic surgery, laparatomy, and rectal extirpation fulfil these criteria. In addition, all patients must be over 18 years of age, fluent German speakers and capable of filling out questionnaires. Further exclusion criteria are: pregnancy, not able to complete a questionnaire, the inclusion in another ongoing clinical trial or submission to a surgical ICU within the last year (this latter criteria renders the impact of an information program less likely).

### Randomization

A block-wise balanced randomization procedure will be used. The allocation to either the intervention or the control group can be found on cards that are sealed into opaque and consecutively numbered envelopes. These envelopes will be opened after the patient has finished the baseline questionnaire. Type of surgical department (heart surgery or visceral, thoracic, vascular surgery) is the only stratification factor and separate piles of envelopes will be prepared for these two groups of patients.

### Blinding of participants

Because of the obvious nature of the intervention patients and field researchers cannot be blinded. Data entry and analysis will be conducted by a neutral researcher who was not involved in data acquisition. Group allocation will be concealed to the analyst until the final analysis of the defined primary and secondary outcomes.

### Study procedure for the individual patient

**Figure 1:**
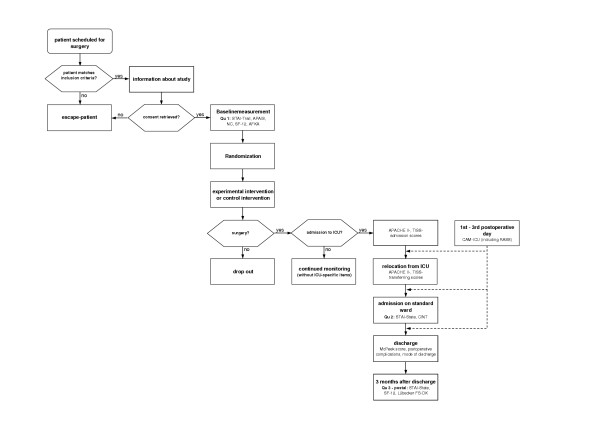
Study procedures for the individual patient.

The study procedure is designed to be compatible with the highly standardized therapy regime of cardiac surgery patients. The single sequences of surgery schedule, surgery, admission on ICU, admission for standard care and discharge are executed due to hospital specific processes (Figure 1). Determination of points of measurement was done according to compiled data of cardiac surgery patients in the Marburg hospital. Patient recruitment is limited to 1 patient per day and department. By the use of a concealed randomized selection procedure with daily assigned random numbers a representative recruiting can be assured.

### Development of the information program

In developing the ICU-specific information program, two main criteria were taken into account: comprehensibility and congruency to informational needs. In addition, empirical and theory based recommendations for patient information were considered [21]. Comprehensibility and congruency were accomplished by using the results of the preliminary studies, additional field observations and informal interviews on the involved wards and finally research literature on the experience of ICU-patients [3,22-32].

A pretest of the program then was carried out to further adapt and fine-tune it. This was done on N = 22 patients with elective ICU-stay [33].

The final program consists of two parts with procedural, sensorial and coping information specifically referring to the ICU-stay (Table 2). The first part is laid down as a standardized text; the verbal information is supplemented by two pictures showing relevant elements of the correspondent ICU. The second part of the program consists of seven cards that show the seven top rated fears associated with ICU-stay as they were named in the pretest. On the backside of the cards are short descriptions of what is done on ICU to counter the feared dangers.

Patients may choose cards that represent their fears and are then presented the fear-rebutting information on the back of the respective card. Furthermore, patients have the opportunity to ask additional open questions. The information program will take about 15 minutes and is given on the day before the scheduled surgery. The intervention is performed by one of the 2 researchers (each with diploma of Nursing and Health science and a registered nurse), who are not involved in the process of daily patient care.

### Measures

#### 1. Self-rated measures

The current emotional patient state is measured with the state-part of the German version of the State-Trait-Anxiety-Inventory (STAI) [34] after readmission to standard care (post-ICU) and 3 months after discharge. At the point of readmission to standard care unit, ICU-related experiences are assessed using the CINT, a scale specifically developed for these purposes and used in preliminary studies of our working group [4,35,36].

Furthermore, health-related quality of life is measured using SF-12 [37] at baseline and 3 months after discharge. Also 3 months post-discharge patient satisfaction is measured using selected items from the Lübecker-Fragebogen-Doppelkarte [38]. Further baseline characteristics that are relevant to our research questions and distinguish between different types of patients are: Trait-Anxiety-Scale [34], preoperative need for information with the Amsterdam Preoperative Anxiety and Information Scale (APAIS) [39], a modified version of the Need-for-Cognition-Scale (NC) [40-43] to measure individual differences in cognitive preoccupation, and finally subjective illness conceptions using assorted items of the Aachener Fragebogen zur Krankheitsattribution (AFKA_I) [44].

#### 2. Observer-rated measures

Postoperative confusion will be monitored on 1^st ^to 3^rd ^postoperative day using the Confusion Assessment Method for the ICU (CAM-ICU) [45,46]. This period was chosen as study results indicate the highest prevalence of postoperative confusion for cardiac surgery patients at these days [47,48]. This instrument is also applicable for mechanically ventilated patients and contains both cognitive and process-related elements. It covers the Richmond Agitation and Sedation Scale (RASS) [49] and 4 delirium characteristics: ,acute onset', ,inattention', ,disorganized thinking' und ,altered level of consciousness'.

As basic trial documentation pre- and post-surgical therapy data is recorded like length of stay, mode of discharge and complications. The Therapeutic Intervention Scoring System (TISS) [50] and the Acute Physiology and Chronic Health Evaluation II (APACHE II) [51] are documented at ICU admission and ICU discharge.

To quantify postoperative recovery the McPeek Recovery Score [52] will be calculated at the end of hospital stay.

Table 1 shows the timetable of the measures during the trial according to the case report form.

Self-rated measures were compiled according to the Total Design Method [53,54] in order to achieve a high degree of study compliance. In addition, a cognitive pretest was executed (N = 3) [55] to verify the properties of the questionnaires by oral interviews. Consequently, the study documentation and the questionnaires were adapted and in the pilot phase of the study a final standard-pretest (N = 5) was done to test responsiveness and feasibility.

### Outcomes

Primary outcome of our study is anxiety as measured by the anxiety-specific part of the CINT questionnaire. This scale relates to experienced anxiety on ICU and includes general anxiety, death related fear, fear of severe suffering, fear of a handicap, fear of the future, fear of uncertainty, panic, strain, depression, loneliness, lack of orientation, uncertainty and anger. All items are rated on a Likert-scale from 1 = never to 4 = always. This measure was specially developed for the reporting of ICU-related experiences and proved very useful in two previous studies [4,35,36].

Secondary outcomes of the study are:

• incidence of postoperative confusion (ICU-CAM) within the first 3 postoperative days

• postoperative state anxiety (STAI) on standard care ward

• postoperative complications, length of stay and McPeek Recovery Score at discharge

• state anxiety (STAI) 3 months follow-up

• quality of life and self-reported health state (SF-12) 3 months follow-up

• patient satisfaction with care and hospital (items from the Lübecker-Fragebogen-Doppelkarte) 3 months follow-up.

### Sample size calculation

Sample size calculation was performed with regard to the anxiety-specific part of the CINT questionnaire as the primary outcome. This score ranges between 0 and 100 with a standard deviation of SD = 17 [36]. Setting alpha at 0.05, beta at 0.20 and Δ = 8.50 (half a standard deviation as a clinically minimal relevant difference [56]) results in a required N = 100 (n = 50 patients in each group) [57]. In order to compensate for possible drop-outs that definitive number of patients to be included into this trial will be set at N = 120. This number relates to patients of the Department of Heart Surgery, a relatively homogenous group of patients.

In order to generalize findings over a wider group of patients, an additional N = 20 from the Department of Visceral, Thoracic and Vascular Surgery will be recruited.

### Drop-outs

Drop-outs will be documented thoroughly and included in data analysis to the point of drop-out. Sensitivity analysis will be undertaken assuming different models of outcome for the drop-outs.

### Data analysis

First of all the whole trial data will be analyzed using descriptive methods: mean, median, standard deviation, frequencies including graphical presentation. Statistical testing will be focused on the cardiac surgery group (n = 120; 60 vs. 60). The main hypothesis representing the primary outcome will be tested with a two-sided t-test for independent samples assuming; the level of statistical significance is set at alpha 0.05 (two-tailed). Secondary outcomes will be tested accordingly.

An important internal analysis will be the correlation of certain individual characteristics to measure which patients benefit more from the information program than others. Especially "need for cognition" and "APAIS" measured at baseline are expected to moderate the impact of the intervention program. This will be analyzed by comparing means of subgroups using the t-test and regression modeling. This is a new study question never investigated in research on preoperative information programs.

Statistical analyses will be performed on an intention to treat basis and will be supplemented using a per protocol approach. The smaller group of patients from the Department of Visceral, Thoracic and Vascular Surgery will be analyzed using descriptive statistics and the main purpose will be to see whether the distribution of crucial outcome parameters resembles the pattern of data found in the cardiac surgery patients.

All analyses will be performed using the SPSS for Windows software.

### Quality assessment

The trial is part of the Nursing Research Network "Mitte-Süd". A report system is established within the network. Quarterly and annual quality reports have to be prepared. These reports undergo peer review and further funding is contingent on a positive review.

### Ethical considerations

The study protocol has been submitted to and approved by the ethical committee of the University Hospital Marburg.

The study relates to a research question that is still unresolved in the literature (equipoise; i.e. it is uncertain whether the intervention or the control group will benefit) and the most rigorous method to settle this issue is the randomized controlled clinical trial. According to the experience in previous trial, participating in a study like this is unlikely to be burdensome for patients [18,4,35,36]. Patients will be fully informed about the purpose of the trial and informed consent is required. All study data is kept locked and anonymous i.e. stored and evaluated using encrypted numbers. The allocation of the patients is documented in a separate reference list that is accessible only to the implementing study personnel.

## Discussion

A measurable, clinically relevant difference in the reduction of anxiety through a preoperative ICU-related information program is expected. Beyond that, interindividual differences in emotional and cognitive preoccupation will be considered that might moderate the impact of information programs and will allow to select patients that may profit most.

## Competing interests

The author(s) declare that they have no competing interests.

## Authors' contributions

AB, SF, MK and TRN contributed to the development of the study protocol. All authors were responsible for the drafting of this paper and approved the final manuscript.

**Table 1:** Points of measurement and clinical outcome measures according to case report form

***points of measurement***	***clinical outcome measures***
baseline (pre-intervention)	STAI-Trait^1^, APAIS^1^, SF-12^1^, NC-items^1^, AFKA_I-items^1^, case report^2^
admission to ICU	TISS^2^, APACHE II^2 ^(admission scores)
1st – 3rd postoperative day a. m.	CAM-ICU (including RASS)^2^
relocation from ICU	TISS^2^, APACHE II^2 ^(transferring scores)
admission on standard ward	STAI-State^1^, CINT-items^1^
discharge from hospital	McPeek recovery score^2^, postoperative complications^2^, length of stay^2^, mode of discharge^2^
3 months after discharge (postal)	STAI-State^1^, SF-12^1^, items from Lübecker FB-DK^1^
	^1^self-rated; ^2^observer-rated

**Table 2:** Topics of the information program

***1) standardized part with information to the following aspects*:**	***2) individualized part with detailed information to the specific fears*:**
- How do you get to the operation theatre and the ICU? - Who is responsible for you on ICU? - What are the devices for? - How does it look like being patient on the ICU? - What is the probable schedule of the ICU-stay? - How can you call attention to your needs? - What else is done for you? - What can you do if you feel stressed?	- fear of complications - fear of feeling locked in - fear of being helpless and dependent on others - fear of pain - fear of suffocation - fear of death or never waking up again - fear of being lonesome

## Pre-publication history

The pre-publication history for this paper can be accessed here:


